# Stent coating containing a charged silane coupling agent that regulates protein adsorption to confer antithrombotic and cell-adhesion properties

**DOI:** 10.1038/s41598-024-65832-5

**Published:** 2024-07-10

**Authors:** Naoki Inuzuka, Yasuhiro Shobayashi, Satoshi Tateshima, Yuya Sato, Yoshio Ohba, Kristina N. Ekdahl, Bo Nilsson, Yuji Teramura

**Affiliations:** 1 R&D Department, Japan Medical Device Startup Incubation Program, 3-7-2 Nihonbashihon-cho, Chuo-ku, Tokyo, 103-0023 Japan; 2 R&D Department, N.B. Medical Inc., 3-7-2 Nihonbashihon-cho, Chuo-ku, Tokyo, 103-0023 Japan; 3 Division of Interventional Neuroradiology, Department of Radiological Sciences, David Geffen School of Medicine, University of California Los Angeles (UCLA), Ronald Reagan UCLA Medical Center, 757 Westwood Plaza, Suite 2129, Los Angeles, CA 90095 USA; 4 Department of Bioengineering, Graduate School of Engineering, The University of Tokyo, 7-3-1 Hongo, Bunkyo-ku, Tokyo, 113-8656 Japan; 5 Cellular and Molecular Biotechnology Research Institute (CMB), National Institute of Advanced Industrial Science and Technology (AIST), AIST Tsukuba Central 5, 1-1-1 Higashi, Tsukuba, Ibaraki 305-8565 Japan; 6 Department of Immunology, Genetics and Pathology (IGP), Uppsala University, Dag Hammarskjölds väg 20, 751 85 Uppsala, Sweden; 7 Master’s/Doctoral Program in Life Science Innovation (T-LSI), University of Tsukuba, 1-1-1 Tennodai, Tsukuba, Ibaraki 305-8577 Japan

**Keywords:** Neurovascular stent, Intracranial aneurysm, Anti-thrombotic coating, Endothelialization, Surface modification, Silane coupling, Biomedical materials, Biomedical engineering

## Abstract

The evolution of endovascular therapies, particularly in the field of intracranial aneurysm treatment, has been truly remarkable and is characterized by the development of various stents. However, ischemic complications related to thrombosis or downstream emboli pose a challenge for the broader clinical application of such stents. Despite advancements in surface modification technologies, an ideal coating that fulfills all the desired requirements, including anti-thrombogenicity and swift endothelialization, has not been available. To address these issues, we investigated a new coating comprising 3-aminopropyltriethoxysilane (APTES) with both anti-thrombogenic and cell-adhesion properties. We assessed the anti-thrombogenic property of the coating using an in vitro blood loop model by evaluating the platelet count and the level of the thrombin–antithrombin (TAT) complex, and investigating thrombus formation on the surface using scanning electron microscopy (SEM). We then assessed endothelial cell adhesion on the metal surfaces. In vitro blood tests revealed that, compared to a bare stent, the coating significantly inhibited platelet reduction and thrombus formation; more human serum albumin spontaneously adhered to the coated surface to block thrombogenic activation in the blood. Cell adhesion tests also indicated a significant increase in the number of cells adhering to the APTES-coated surfaces compared to the numbers adhering to either the bare stent or the stent coated with an anti-fouling phospholipid polymer. Finally, we performed an in vivo safety test by implanting coated stents into the internal thoracic arteries and ascending pharyngeal arteries of minipigs, and subsequently assessing the health status and vessel patency of the arteries by angiography over the course of 1 week. We found that there were no adverse effects on the pigs and the vascular lumens of their vessels were well maintained in the group with APTES-coated stents. Therefore, our new coating exhibited both high anti-thrombogenicity and cell-adhesion properties, which fulfill the requirements of an implantable stent.

## Introduction

Minimally invasive endovascular interventions have gained widespread acceptance in the rapidly evolving landscape of medical device technology and they have revolutionized the treatment of conditions such as intracranial aneurysms. The use of endovascular treatments has increased steadily since the introduction of aneurysm coils in the 1990s. By 2004, the approach used to treat unruptured intracranial aneurysms had predominantly shifted to endovascular procedures; nowadays, approximately 70% of such aneurysms are treated within the blood vessel, reversing the ratio from open surgical clipping^1,2^. This shift towards endovascular interventions, which are less invasive and reduce the patient burden, has led to quicker societal reintegration post-treatment.

Despite progress in the development of endovascular interventions and their widespread implementation, thrombotic complications on the surfaces of metal implants such as stents and coils persist. The rate of thromboembolic complications following stent placement ranges from 5 to 15%^3,4^. Numerous coating technologies have been explored to overcome these challenges^5–7^. Although coatings such as poly(2-methacryloyloxyethyl phosphorylcholine)-based polymer (MPC polymer), poly(2-methoxyethylacrylate) (PMEA), and heparin are promising in terms of anti-thrombogenicity, and have been integrated into clinical practice^8–10^, they have yet to meet all the desired attributes, particularly simultaneous anti-thrombotic efficacy and the swift integration of the metal implant, i.e., endothelialization.

For instance, despite its high antithrombotic properties, MPC polymer hinders endothelial cell adhesion^11^ and PMEA-coated devices have a higher rate of thrombotic complications than MPC polymer-coated devices^9,10^. An ideal coating would offer reliable anti-thrombotic properties, promote endothelialization, be sourced from non-biological materials, and minimize the potential for hemorrhagic issues.

Previous efforts have largely focused on preventing non-specific protein adsorption using MPC polymer, PMEA or regulating the coagulation cascade with heparin. These initiatives, while significant, have not fully satisfied the multifaceted requirements outlined above. Therefore, researchers have sought new solutions. We have investigated various coatings comprising small synthetic compounds and polymers for improving blood compatibility and endothelialization, and have identified some candidates. Herein, we demonstrate the potential of coatings comprising 3-aminopropyltriethoxysilane (APTES) for intravascular stents. APTES is used to modify metal, silicon wafer, or glass surfaces to introduce amine groups via silane coupling, and also utilized for functionalization with polymer coating on the surface. The introduced amino group contributed to the stable bonding between the substrate and the polymer^12,13^. Here we coated intravascular stents with APTES and investigated their blood compatibility using the Chandler loop model with human whole blood by measuring the platelet count and monitoring a coagulation marker. We also used scanning electron microscopy (SEM) to investigate the coated surfaces and the proteins adsorbed onto them following exposure to human blood. In addition, we conducted a cell-binding assay on the coated surfaces using primary endothelial cells. Finally, we carried out in vivo safety evaluations for 1 week after implanting the coated stents into pig vessels.

## Materials and methods

### Sample preparation

#### Coating a stent and a flat plate of nickel–titanium alloy with APTES and MPC polymer

A self-expanding stent was constructed by laser-cutting a nickel–titanium alloy tube, which was then heat-treated to induce shape memory and electropolished to ensure a smooth surface by our company (NB-stent-01). The coating was produced by dissolving APTES (Shin-Etsu Chemical Co., Ltd, Tokyo, Japan) in ethanol (EtOH; 5% (v/v))^14^. The stent was exposed to UV ozone irradiation for 15 min using a UV ozone generator (SKB401Y, San Energy Co., Ltd, Tokyo, Japan). The treated stent was then immersed in the prepared coating solution for 2 h. Subsequently, the stent was thoroughly rinsed with EtOH to eliminate residual chemicals and dried in a vacuum at 70 °C for 30 min. As a control, a similar stent was coated with MPC polymer (Lipidure 3001, NOF Co., Tokyo, Japan) according to the protocol provided by the company. Briefly, the MPC polymer was dissolved in EtOH (4 wt%). The resulting solution was then mixed with an equal volume of 0.1 mM hydrochloric acid while stirring continuously for 30 min. The stent was then exposed to UV ozone irradiation for 15 min using the UV ozone generator and immersed in the MPC polymer solution for 1 min. After removal from the MPC polymer solution, the stent was dried at 80 °C for 1 h, then rinsed with distilled water to remove any residual polymer. APTES and MPC polymer were also applied to a flat nickel–titanium alloy plate of the same composition as the stent using the same coating procedure.

#### Coating of silica beads with APTES

Silica beads were also coated with APTES using the same method. The silica beads (Sicastar #43-00-1003, Micromod GmbH, Rostock, Germany) were exposed to UV ozone irradiation for 15 min using a UV ozone generator. The treated beads were then immersed in the APTES coating solution and shaken for 2 h at room temperature. After the reaction, the beads were washed with ethanol and further rinsed twice with phosphate-buffered saline (PBS) by subjecting them to centrifugation (× 100 *g*, 10 min, room temperature).

### X-ray photoelectron spectroscopy (XPS)

The surface composition of the coated stents was analyzed by X-ray photoelectron spectroscopy (XPS) using a KRATOS ULTRA2 instrument (Shimadzu Co., Kyoto, Japan). The take-off angle was set to 90° and the operational pressure was maintained below 1.0 × 10^−7^ Pa. The binding energies were calibrated using the C1*s* peak attributable to the alkyl groups at 285.0 keV. The elemental composition of the surface was determined by quantifying the areas under the individual peaks.

### Blood compatibility assay using the Chandler loop model

Fresh human whole blood was obtained from healthy volunteers following approval from the ethics committee of the Japan Medical Start-up Incubation Program (JMPR), under research protocol JMPR-EMPR-220607-01. In addition, all procedures were performed in accordance with the guidelines and regulations approved by the ethics committee. Informed consent was obtained from all participants in the study.

The blood was collected in an anticoagulant-free system and immediately treated with heparin (Mochida Pharmaceutical Co., Ltd, Tokyo, Japan) to obtain a final concentration of 1.0 IU/mL. All samples were transferred to specified incubation vials within 10 min of collection.

The Chandler loop model, which was previously established in our laboratory^15^, was utilized to evaluate the blood compatibility of the coated stents^16–18^. The Chandler loop model was accepted for the evaluation of blood compatibility of artificial materials in vitro as seen in ISO10993-4 Biological evaluation of medical devices -Part 4: Selection of tests for interactions with blood/Annex A Preclinical evaluation of cardiovascular devices and prostheses. This model comprises a polyurethane tube (60 cm long, internal diameter of 4.0 mm) and a plastic connector, which were treated with MPC polymer (Lipidure CM5206, NOF Co., Tokyo, Japan). Briefly, the inside of the loop was filled with MPC polymer solution (0.5% in ethanol) and rotated at 10 rpm for 3 h. The excess solution was then removed, and the loop was dried in air.

The stent was inserted into the tube and positioned at the opposite end from the plastic connector when the tube was closed. Heparin-treated blood (2 mL) was carefully poured into the tube while ensuring no air bubbles formed. Both ends of the tube were then sealed with a connector to create a closed loop, which was mounted on a rotating platform and rotated at 10 rpm for 3 h at a controlled temperature of 37 °C (shear stress: 0.043 dyn/cm^2^). Subsequently, the stent and blood were separately collected for analysis. The stent was gently rinsed with physiological saline and preserved in 4% paraformaldehyde buffered with phosphate. The blood was collected and mixed with ethylenediaminetetraacetic acid (Thermo Fisher Scientific, Inc., Grand Island, USA) to obtain a final concentration of 0.01 M. The platelets were then counted (pocH80i, Sysmex Corporation, Kobe, Japan). We also measured the initial platelet count to calculate the consumption ratio of the remaining platelets during incubation for 3 h. Finally, the whole blood was centrifuged (× 2500*g*, 10 min, 4 °C) to collect the plasma, which was stored at − 80 °C until required.

To evaluate the coagulation marker, the concentration of the thrombin–antithrombin complex (TAT) in plasma was evaluated using a sandwich enzyme-linked immunosorbent assay (ELISA) kit (ASSAYPRO LLC, MO, USA). The ELISA was conducted without diluting the collected plasma.

### Scanning electron microscopy (SEM)

The stents were exposed to blood for 3 h in the Chandler loop. They were then collected and rinsed with physiological saline. The stents were then incubated overnight in 4% paraformaldehyde and air-dried at room temperature for 24 h prior to SEM examination. The stents were investigated using a TM4000PlusII scanning electron microscope (Hitachi High-Tech, Tokyo, Japan). Each stent was imaged at 20 different locations, which were randomly chosen. We performed a semi-quantitative evaluation based on the criteria delineated in Table 1.

**Table 1:** Evaluation criteria for thrombotic scores.

Score	Evaluation criteria
Level 1	Almost no thrombus formation on the stent surface, which remains flat
Level 2	Slight thrombus formation on the stent surface, but mostly flat
Level 3	Thin thrombus formation observed in localized areas of the stent surface
Level 4	Wide areas of the stent surface covered with a thin thrombus, but overall flat
Level 5	Most of the stent surface is flat, but localized raised thrombus is observed
Level 6	Wide areas of the stent surface covered with a thin thrombus, with localized raised thrombus
Level 7	Most of the stent surface covered with a thin thrombus, with some areas having raised thrombus
Level 8	Entire stent surface covered with thrombus, with numerous localized raised thrombi
Level 9	Entire stent surface covered with thick raised thrombus, with some areas having very thick multilayered thrombus
Level 10	Entire stent surface completely covered with very thick, multilayered thrombus

### Protein adsorption studies using a flat nickel–titanium alloy plate coated with APTES

#### Quantification of adsorbed total protein

We prepared flat nickel–titanium alloy plates (10 mm × 5 mm) coated with APTES for the protein adsorption studies because the surface areas of the stents were insufficient for this purpose. The flat plates with or without the APTES coating were incubated in human plasma for 2 h at room temperature, then washed with Milli-Q water to remove unbound proteins. To detach the proteins from its surface, each plate was immersed in a 10 mg/mL sodium dodecyl sulfate solution, vigorously rotated for 30 min, and subjected to ultrasonication for 5 min. The resulting supernatant was collected and the total protein concentration was determined using a Micro BCA™ protein assay (Thermo Fisher Scientific, Inc., Grand Island, USA).

#### Quantification of adsorbed human serum albumin (HSA)

We quantified the amount of HSA adsorbed on the flat nickel–titanium alloy plates after exposure to human plasma using anti-HSA antibodies. An MK132 Human Albumin EIA Kit (Takara Bio Inc., Shiga) was used to quantify albumin adsorption. The APTES-coated and uncoated plates were incubated in human plasma for 2 h, then washed three times with PBS. Subsequently, the plates were incubated in 4% Block Ace (KAC Co., Ltd, Kyoto, Japan) for 30 min. The plates were then exposed to a threefold diluted horseradish peroxidase-labeled anti-HSA antibody solution and allowed to react at room temperature for 1 h. The Substrate Solution was then added and incubation was continued for a further 15 min at room temperature. After adding the Stop Solution and mixing, the samples were aliquoted into a 96-well plate for measurement. Zero adjustment was performed using distilled water as a control, and absorbance was measured at a wavelength of 450 nm. To determine the change in albumin adsorption, the absorbance of the bare plates was set to 100% in each experiment. The results are expressed as a percentage relative to this baseline.

### Measurement of the zeta potential of coated silica beads

Zeta potential measurements were performed on uncoated and APTES-coated silica beads using a Zetasizer Nano ZS instrument (Malvern Instruments Co., Malvern, UK). The beads were washed twice with PBS and centrifuged (×100*g*, 10 min, room temperature). They were then incubated in human plasma for 2 h. Finally, the beads were again washed twice with PBS and centrifuged (×100*g*, 10 min, room temperature) in preparation for the zeta potential measurements.

### In vitro cell adhesion assay

Human umbilical vein endothelial cells (HUVECs; Lonza, Basel, Switzerland) were used to evaluate cell adhesion on coated metal plates. The initially frozen HUVECs were cultured for 3 days until they reached semi-confluence. Cells adhering to the metal substrate were labeled with CellTrace Oregon Green 488 (Thermo Fisher Scientific, MA, USA), adjusted to a final concentration of 2 µM in the culture medium, and incubated for 30 min. After trypsinization at 37 °C for 3 min, unreacted fluorescent dye was removed by centrifugation at 220 g for 5 min. The coated metal plates were placed in a 24-well plate, and the fluorescently labeled cells were seeded at a density of 300,000 cells per well. After incubation for 24 h in an incubator containing 5% CO_2_ at 37 °C, the metal surfaces were examined for fluorescence using an upright fluorescence microscope (BX53-44-FL-1, Olympus, Tokyo, Japan). The obtained images were processed using ImageJ software. Fluorescence images of each metal plate were obtained and the area of fluorescence was quantified. The average area of adhered cells was calculated based on measurements from five different wells (n = 5) for each condition.

### In vivo implantation of coated stents into pigs

Self-expanding stents coated with APTES were sterilized with ethylene oxide gas and implanted into healthy Göttingen minipigs weighing 20–30 kg. The animal experiments were conducted at the Shuzenji Branch of the Nihon Bioresearch Inc. Hashima Laboratory. These experiments adhered to the “Basic Guidelines for the Implementation of Animal Experiments in Institutions under the Jurisdiction of the Ministry of Health, Labour, and Welfare” as notified by the Japanese government, and were approved by the Animal Experiment Committee of the facility.

Pre-quarantined and acclimatized pigs were housed individually in stainless steel cages under controlled environmental conditions: 16–27 °C and a 12-h light–dark cycle. The pigs were administered clopidogrel (75 mg/body) and aspirin (200 mg/body) via mixed feed, starting 3 days before stent placement and continuing until the day of necropsy. Two male pigs were used, and the internal thoracic artery and ascending pharyngeal artery were selected as the stent placement vessels. The vessel for the animal experiments was chosen based on several critical criteria. First, the diameter for our stent ranges from 2.0 to 3.0 mm, which matches the diameter of the chosen vessel. Additionally, the vessel location is anatomically favorable for the access with extraction, so that the experimental procedure could be easy. Lastly, considering the intended use of the stent in cerebral vessels, it was imperative to select a location less affected by physical movements, ensuring consistent conditions throughout the experiment with different recipients. A mixture of atropine sulfate (0.05 mg/kg, 0.1 mL/kg), medetomidine hydrochloride (0.05 mg/kg, 0.05 mL/kg), and midazolam (0.25 mg/kg, 0.05 mL/kg) was administered intramuscularly into the base of the ear for preoperative sedation. Heparin was administered at a rate of 3 mL/head before the procedure, and the activated clotting time was measured using an Actalyke MINI II system (TriTech Inc.). Subsequent doses of 1 mL/head were administered intravenously into the auricular vein every 1–2 h. Stents coated with APTES or MPC polymer were placed in the respective left and right vessels using a Headway 21 microcatheter (Terumo MicroVention, Tokyo, Japan). Postoperative care included administering atipamezole hydrochloride (0.25 mg/kg, 0.05 mL/kg) intramuscularly into the base of the ear to awaken the pig, which was subsequently supplied with oxygen only until fully awake. Ampicillin sodium (1000 mg/body, 5 mL/body) was administered intramuscularly into the base of the ear for 3 days, including the day of the procedure. Buprenorphine hydrochloride (0.01 mg/kg, 0.05 mL/kg, calculated from the weight on the day of the procedure) was administered intramuscularly into the base of the ear for three days, including the day of the procedure (twice on the day of the procedure and once on subsequent days). A guiding catheter was inserted up to the proximal part of the target vessel on the 7th day after stent implantation, and angiography (ARCADIS GEN2, Siemens, Germany) was performed to assess the vascular condition.

The animals were sacrificed with appropriate way and include anesthesia agent used and euthanasia method, which were approved in “Basic Guidelines for the Implementation of Animal Experiments in Institutions under the Jurisdiction of the Ministry of Health, Labour, and Welfare”, which is equivalent to ARRIVE guidelines. Minipigs were deeply anesthetized through an intravenous injection of thiopental sodium (dose: 25 mg/kg, volume: 1 mL/kg) into the auricular vein. Following anesthesia, euthanasia was achieved by exsanguination from the abdominal aorta.

### Statistical analysis

Initial platelet count values were normalized to account for individual variations. Thrombosis scores were assigned based on images captured at 20 different locations for each sample and the standard error was calculated for each score. Statistical analyses were performed using GraphPad Prism 10 software (GraphPad Software, San Diego, CA, USA). One-way analysis of variance (ANOVA) was followed by post hoc comparisons utilizing Tukey’s multiple comparison test to ascertain the statistical significance of each result.

## Results

### XPS analysis

XPS analysis confirmed the surface modifications of the stents following coating with APTES. We evaluated three coated stents by obtaining two measurements from each to check the uniformity of the coating process. The elemental composition ratios presented in Table 2 were averaged across all measurements. Silicon (Si) was detected at a level of 2.4% ± 0.3% on the APTES-coated stents, verifying the immobilization of APTES on the stent surfaces.

**Table 2:** Atomic composition percentages of the surface elements, as quantified by X-ray photoelectron spectroscopy (XPS).

Sample	Atomic component (%)					
	Si (2*p*)	C (1*s*)	N (1*s*)	Ti (2*p*)	O (1*s*)	Ni (2*p*3/2)
Bare (n = 2)						
Mean	0.0	34.9	0.2	15.5	46.7	2.6
SD	0.0	1.3	0.3	1.6	0.1	0.0
APTES (n = 6)						
Mean	2.4	40.7	1.8	13.0	40.9	1.1
SD	0.3	4.9	0.4	2.0	2.4	0.7

(APTES = 3-aminopropyltriethoxysilane).

### In vitro evaluation: whole blood contacting test in the Chandler loops

**Figure 1:**
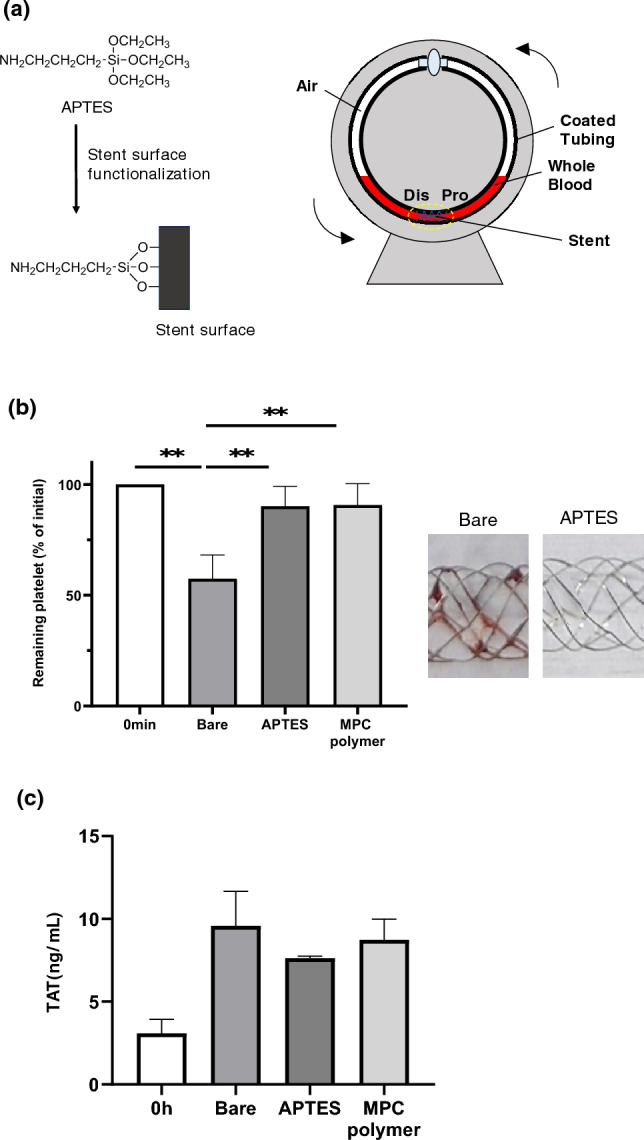
Blood compatibility study using Chandler loop model. (**a**) Chemical structure of APTES and the functionalization of stent surface. A schematic representation of the stent–blood contact test using the Chandler loop model. In this model, 2 mL of blood is introduced into the tube, with the remaining volume filled with air. The stent is positioned opposite the connector within the tube. (**b**) Results of platelet counts following the blood contact experiment utilizing the Chandler loop model. The initial platelet count, taken immediately after blood sampling, was set as the baseline (100%). The figure shows the percentage of platelets remaining in the blood after 3 h of contact with the stent. Blood samples from three different experiments (n = 3) were analyzed, and the data are expressed as the mean percentage ± standard deviation (SD). The graph indicates that both the 3-aminopropyltriethoxysilane (APTES)- and the poly(2-methacryloyloxyethyl phosphorylcholine)-based polymer (MPC polymer)-coated surfaces significantly inhibited the reduction of platelets compared to the bare stents, as demonstrated by the statistically significant preservation of platelet counts (**p < 0.01). The pictures of bare stent and APTES-coated stent are taken at 3 h incubation in whole blood after wash with saline. Clots were attached on the bare stent while no clots were observed on the APTES-coated stent. (**c**) Results of thrombin–antithrombin (TAT) concentration measurements in blood samples retrieved after the Chandler loop model experiment. The baseline of TAT concentration was established using values obtained immediately after blood collection. A comparison of these baseline values revealed a significant increase in TAT concentration across all stent conditions post-experiment. However, when comparing the different stent conditions to each other, no significant differences in TAT concentration were observed. The measurements were taken from blood samples from three different experiments (n = 3), with data expressed as mean ± standard deviation (SD).

**Figure 2:**
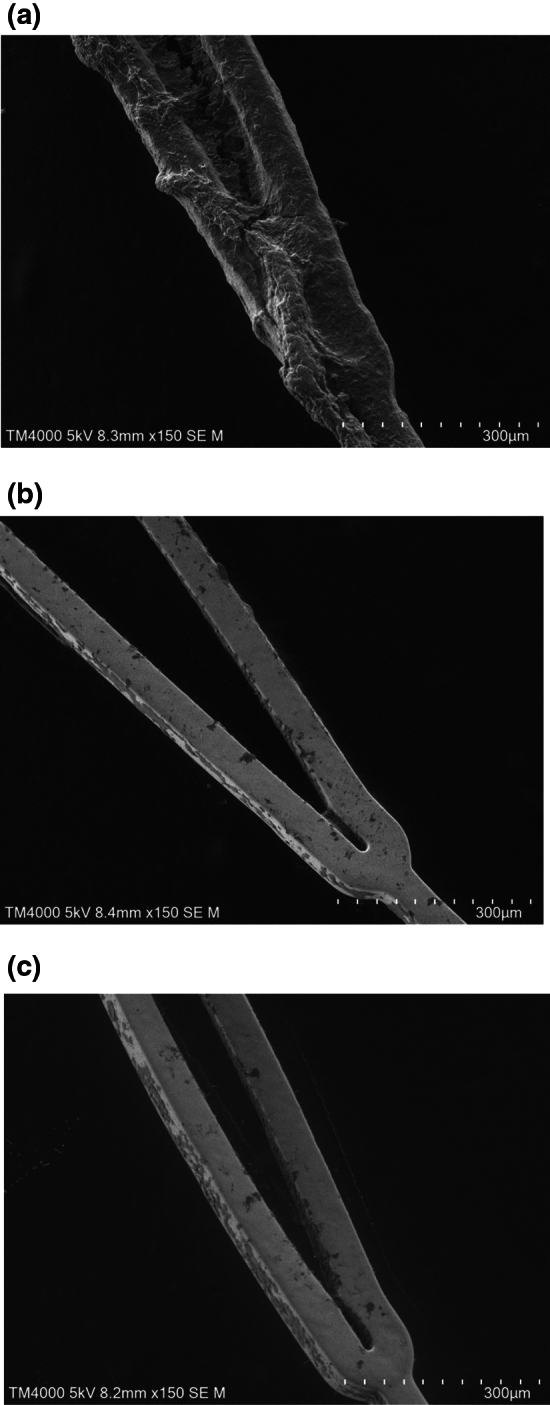
Representative scanning electron microscopy (SEM) images of three different stent samples following a blood contact period of 3 h in the Chandler loop model. (**a**) Corresponds to a stent without any coating, (**b**) to a stent coated with 3-aminopropyltriethoxysilane (APTES), and (**c**) to a stent coated with poly(2-methacryloyloxyethyl phosphorylcholine)-based polymer (MPC polymer).

**Figure 3:**
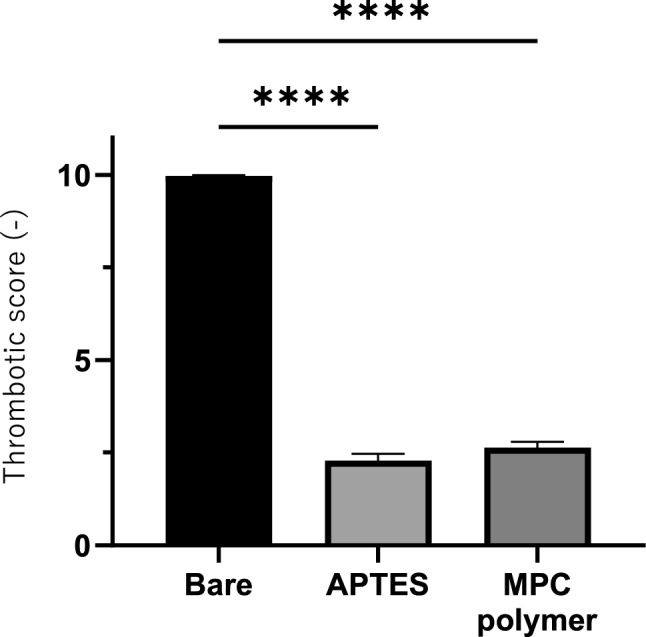
Thrombogenicity scoring of stents derived from scanning electron microscopy (SEM) imaging. For each stent, SEM images were captured at 20 predefined locations, ensuring comprehensive coverage. This involved taking shots at intervals of 1.5 mm along the stent's axial direction and 1 mm along the diameter direction. Scores were assigned to each stent at 20 distinct locations based on the criteria established in Table 1. A total of 3 stents (n = 3) were evaluated, amounting to 60 separate scoring assessments. The graph presents the mean thrombogenicity score with standard error of the mean. Both the 3-aminopropyltriethoxysilane (APTES)- and poly(2-methacryloyloxyethyl phosphorylcholine)-based polymer (MPC polymer)-coated surfaces had significantly reduced thrombogenicity scores compared to the bare stents, indicating improved antithrombotic performance (****p < 0.001).

In vitro Chandler loop whole blood contact tests (Fig. 1a) were used to evaluate the efficacy of the APTES coating on the stents with regard to platelet adhesion. With a bare stent, the number of platelets in the whole blood decreased to approximately 57% of the initial platelet count. In contrast, with an APTES-coated stent the number of platelets in the whole blood decreased to an average of 90.2% ± 8.9% of the initial platelet count. The results for the stents coated with MPC polymer were similar; the number of platelets in the whole blood decreased to an average of 90.7% ± 9.7% of the initial platelet count. The higher platelet retention of the APTES-coated stents was statistically significant (*p* < 0.01), as shown in Fig. 1. There was no significant difference in TAT generation among the various stents; however, the APTES-coated stents tended to suppress TAT generation (Fig. 1c). The retrieved stents were examined by SEM at 20 distinct points and representative images are displayed in Fig. 2. The images were scored according to the criteria set out in Table 1 and the average score (± standard error of the mean) was calculated (Fig. 3). The bare stents frequently formed thick and numerous thrombi. In contrast, the APTES-coated stents demonstrated a significant reduction in thrombus formation.

### Zeta potential

**Figure 4:**
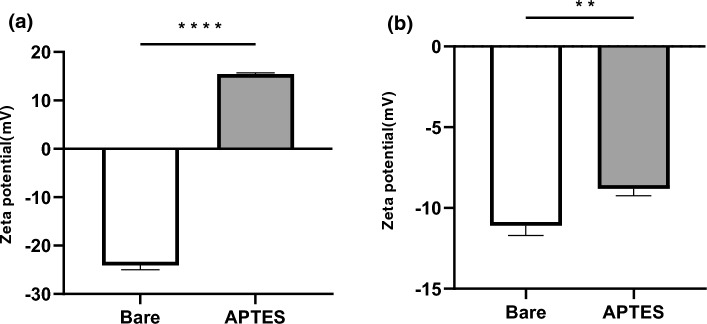
Zeta potential analysis of APTES-treated surface. (**a**) Depicts the zeta potential of silica beads post-treatment, measured in phosphate-buffered saline (PBS) and demonstrating a statistically significant alteration compared to the surface with no coating (****p < 0.001), with each group consisting of nine measurements (n = 9) and data expressed as mean ± standard deviation (mean ± SD). (**b**) Illustrates the zeta potential of the same silica beads following immersion in plasma and subsequent rinsing, confirming a statistically significant difference (**p < 0.01) with each group consisting of nine measurements (n = 9) and data expressed as mean ± standard deviation (mean ± SD).

The zeta potential measurements in PBS revealed the impact of the APTES coating on the surface charge properties of the stents. Initially, the uncoated silica beads had a zeta potential of − 24.1 ± 0.9 mV. After coating with APTES, this value increased significantly to 15.4 ± 0.3 mV (****p < 0.001, Fig. 4a). The silica beads were immersed in plasma and subsequently washed with PBS before the measurements were obtained. The zeta potential of the uncoated beads was − 11.1 ± 0.6 mV, whereas that of the APTES-coated beads was − 8.8 ± 0.4 mV, indicating a less negative surface charge after coating (*p* < 0.01, Fig. 4b). These findings indicate a change in the protein adsorption profile due to the coating.

### Analysis of the adsorbed proteins on the coated stents

**Figure 5:**
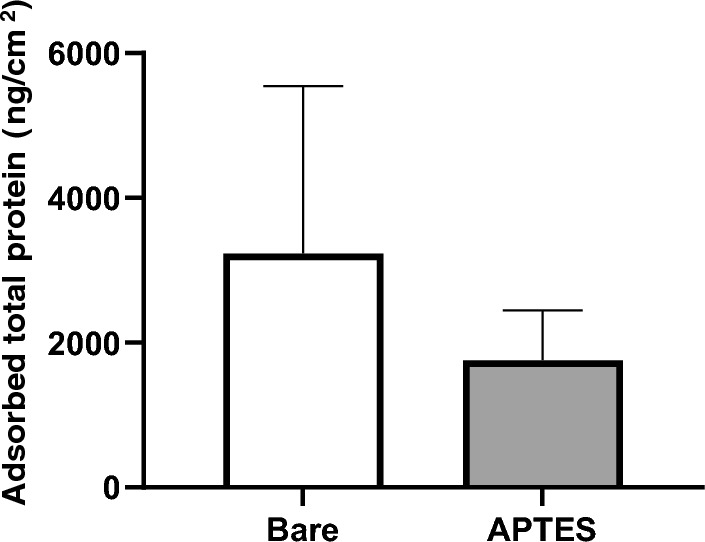
Quantitative comparison of total protein adsorbed on the surface. Nine independent measurements (n = 9) were taken for each group, with data presented as mean ± standard deviation (mean ± SD). There was no significant difference between the groups. To detach the proteins from its surface, each plate was immersed in a 10 mg/mL sodium dodecyl sulfate solution, vigorously rotated for 30 min, and subjected to ultrasonication for 5 min. The total protein concentration in the supernatant was determined using a Micro BCA™ protein assay.

We quantified the adsorption of total proteins on nickel–titanium flat plates submerged in plasma. The uncoated plates had a mean protein mass per unit area of 3231 ± 2312 ng/cm^2^. In contrast, the plates coated with APTES had a lower average protein mass per unit area of 1757 ± 690 ng/cm^2^ (Fig. 5). Although there was no statistical significance, the results suggest a tendency towards reduced total protein adsorption attributable to the APTES coating.

**Figure 6:**
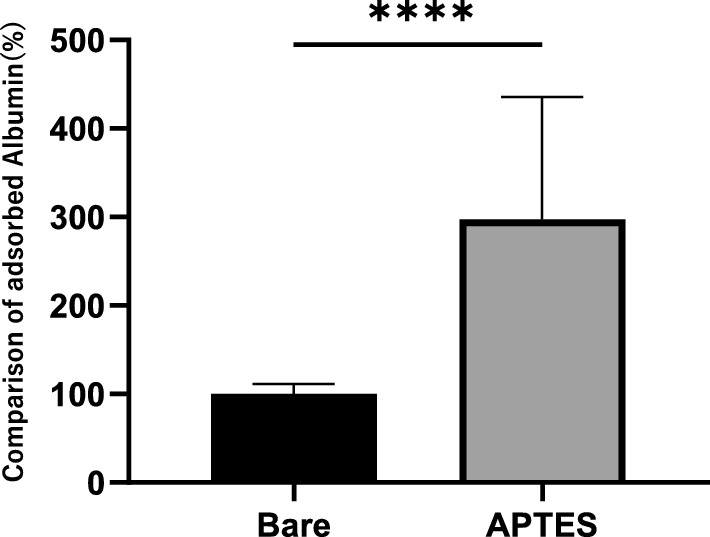
Detection and quantification of albumin adsorption on the surfaces. Albumin adsorption on the surface with no coating was set as the reference baseline, and the data are expressed as a percentage ratio. Measurements were conducted with nine replicates (n = 9), and the results are presented as the mean ± standard deviation (mean ± SD). There was a significant difference between the surfaces: the coated surfaces demonstrated increased albumin adsorption (****p < 0.001).

Analysis of the plasma-immersed nickel–titanium flat plates revealed that the specimens coated with APTES exhibited a statistically significant increase in albumin adsorption: the adsorption rate soared to 298% ± 138% compared to that of the uncoated baseline (****p < 0.001, Fig. 6). This significant uptick indicates that the APTES coating selectively increases albumin binding, despite an overall trend of reduced protein adherence.

### In vitro evaluation of endothelial cell adhesion

**Figure 7:**
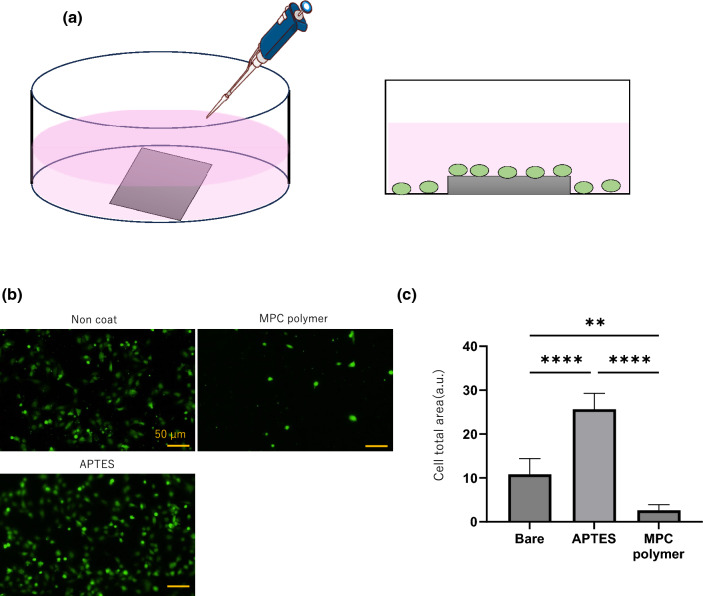
Endothelial cell-binding test on treated metal surface. (**a**) A schematic diagram illustrating the setup for the in vitro cell adhesion assay. In this assay, surface-treated metal plates were placed into each well of a 24-well plate. Fluorescently labeled cells were then seeded into each well at a density of 300,000 cells per well. Following incubation for 24 h, the cells that had adhered to the metal plates were examined and assessed using a fluorescence microscope. (**b**) Assessment of the adhesion of human umbilical vein endothelial cells (HUVEC) on the various surfaces, visualized using fluorescent staining and confirmed by fluorescent microscopy. Figure 6c quantifies the area covered by the adherent cells based on the fluorescence emitted. Five independent experiments (n = 5) were conducted for this analysis, with data expressed as the mean ± standard deviation (SD). The 3-aminopropyltriethoxysilane (APTES)-coated surfaces significantly increased cell adhesion compared to both the bare and poly(2-methacryloyloxyethyl phosphorylcholine)-based polymer (MPC polymer)-coated surfaces (****p < 0.001). Conversely, compared to the bare surfaces, the MPC polymer-coated surfaces significantly reduced cell adhesion (**p < 0.01).

We seeded fluorescently stained HUVECs onto each metal substrate in culture dish (Fig. 7a). Representative images of the HUVECs adhering to each surface are shown in Fig. 7b. The area of fluorescence was measured to quantify the total area of cell adhesion by image processing. The area of adherent cells emitting fluorescence was 11 ± 4 arbitrary units (a.u.) on bare substrates, 26 ± 4 a.u. on APTES-coated substrates, and 3 ± 1 a.u. on MPC polymer-coated substrates (Fig. 7c). The area of cells adhering to APTES-coated substrates was significantly larger than that adhering to the bare or MPC polymer-coated substrates (*p* < 0.001). Although some cells adhered to the MPC polymer-coated substrates, the area of adherent cells was significantly reduced compared to that on the bare substrates (*p* < 0.01), indicating that the MPC polymer prevents protein adsorption and subsequent cell adhesion^6^. We found a significant increase in cell adhesion on the APTES-coated substrates, indicating that APTES does not inhibit endothelialization, but rather promotes it. This is presumably because the surface was positively charged, so protein adsorption and cell attachment occurred rapidly^19,20^.

### In vivo acute safety testing of the APTES-coated stent using minipigs

**Figure 8:**
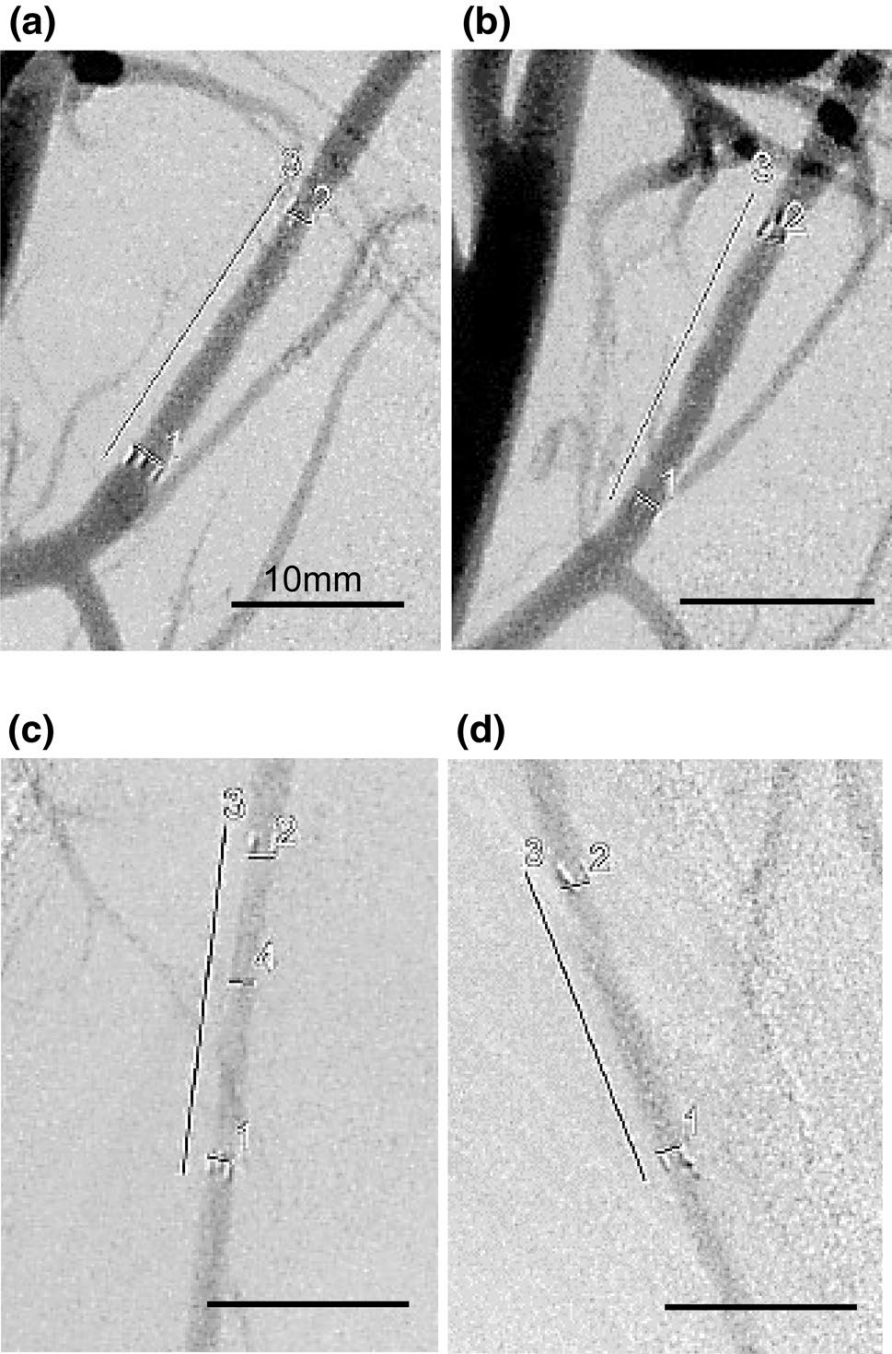
Representative angiographic images obtained 1 week after stent implantation in each blood vessel during the animal experiment. These angiograms visually confirm the maintenance of the vascular lumen and blood flow, with no changes in vessel diameter observed before and after stent placement. The inner lumen of the stents remained intact, with no alterations in the stent's internal diameter. Blood flow was consistently maintained post-implantation, indicating no adverse effects due to the stenting. Furthermore, the general condition of the pigs remained stable and normal, with no observable changes pre- and post-stent implantation. Ascending pharyngeal artery with stent placement (**a**: 3-aminopropyltriethoxysilane (APTES)-coated, **b**: poly(2-methacryloyloxyethyl phosphorylcholine)-based polymer (MPC polymer-coated). Internal thoracic artery with stent placement (**c**: APTES-coated, d: MPC polymer-coated).

We assessed the safety of the coated stent by determining the patency of the vascular lumen and the integrity of blood flow in the ascending pharyngeal artery and internal mammary artery following stent insertion by examining the angiographic images shown in Fig. 8a–d. The APTES-coated stent was implanted in normal, non-aneurysmal blood vessels. This approach is commonly used in safety evaluation studies because it enables the assessment of the stent’s performance in a standard vascular environment.

Herein, we implanted both APTES- and MPC polymer-coated stents into minipigs. MPC polymer-coated stents are used clinically owing to their anti-fouling property. Therefore, we used the MPC polymer-coated stent as a control. We found that blood flow was maintained well and we could not detect any distal embolization during the week following implantation of either the APTES- or MPC polymer-coated stents. There were no changes in the vessel diameter pre- and post-stent implantation in either case. The inner lumen of the stents remained constant, with no alterations in the internal diameter observed, ensuring the integrity of the stent placement. Additionally, the blood flow remained stable and unchanged post-implantation. Furthermore, the overall health condition of the pigs remained normal throughout the experiment, with no adverse effects observed pre- and post-stent implantation. The minipigs exhibited no systemic abnormalities, indicating that the acute safety profile was well guaranteed. In this in vivo experiment, the acute safety of the APTES-coated stent was evaluated by comparing it to that of the MPC polymer-coated stent, which has an established safety profile in clinical settings^21,22^. The results indicate that the safety of an APTES-coated stent in the acute phase is comparable to that of an MPC polymer-coated stent. Therefore, we verified the safety of the APTES coating on stents in the acute phase after implantation.

## Discussion

Our study demonstrated that the APTES coating is highly anti-thrombogenic and has the ability to interact with endothelial cells. Our coating modulates protein adsorption in human blood, particularly by enhancing HSA adsorption, as determined by our analyses. Generally, when metal or plastic is exposed to blood, nonspecific protein adsorption occurs rapidly on its surface. Because the early phase of nonspecific protein adsorption is a critical step in thrombus formation, it is important to control initial protein adsorption to regulate the anti-thrombogenic properties of the surface by coating with anti-fouling polymers and heparin derivatives^5–7,15,23^. Regarding the nonspecific adsorption of proteins onto material surfaces in blood, the adsorption of HSA is limited. This does not reflect its abundance in the blood, i.e., a concentration of up to approximately 4.5 wt%^24,25^. In contrast, fibrinogen, which is present in the blood at a lower concentration (approximately 0.2 wt%), positively adheres to surfaces, regardless of its concentration in the bloodstream^26^. This leads to platelet adhesion and subsequent thrombus formation due to interaction with the adsorbed fibrinogen^27^.

In the present study, the surfaces coated with APTES exhibited an increase in HSA adsorption compared to that of the bare surfaces, indicating the rapid recruitment of more HSA from the bloodstream. Moreover, when the APTES-coated surfaces came into contact with human blood, they had a more neutral charge than the bare surfaces, so the total amount of protein adsorbed by them was lower. Proteins immediately adhere to surfaces within seconds and subsequently cover the whole surface before blood cells approach the surface when in contact with blood^28,29^. Therefore, our results suggest that HSA was preferentially adsorbed onto the APTES-coated stent surface, which was able to block the adsorption of other coagulation-related proteins as well as platelets.

APTES has a primary amine group. Therefore, the outermost layer of the coated metal becomes enriched with NH_3_^+^ groups under physiological conditions. Zeta potential measurements revealed that APTES-coated surfaces were positively charged in contrast to the negatively charged bare surfaces. This difference plays a pivotal role in protein adsorption, during which serum proteins with opposite charges strongly interact with the surface^30^. In particular, we consider that HSA, which is a negatively charged protein, adheres rapidly and forms a stable protein layer on the positively charged APTES-coated surfaces. This stable HSA layer can reduce the exchange adsorption of coagulation-related proteins, resulting in higher blood compatibility, as corroborated by the blood loop experiments and in vivo pig studies.

The adsorbed proteins on the surface might undergo exchange with other proteins. This is known as the Vroman effect, whereby coagulation proteins such as high-molecular-weight kininogen and factor XII are eventually adsorbed and replace the initially adsorbed proteins^31^. Although abundant proteins such as HSA initially adhere to the surface, they are gradually replaced by coagulation proteins, which are more reactive with the surface, over time. Therefore, the surface becomes covered with coagulation proteins such as high-molecular-weight kininogen and factor XII^32^. It is possible for the surface state to change over time. This may trigger a coagulation cascade. However, our treated surface was able to maintain the initial HSA layer so that the anti-thrombogenic property was retained, as corroborated by the week-long in vivo pig study and the 3 h Chandler loop experiments.

It has been reported that pre-coating with albumin confers anti-thrombogenic properties and does not inhibit the adhesion of endothelial cells^26,27^. Therefore, surfaces are treated with albumin as a blocking reagent before implantation to inhibit non-specific protein adsorption. Although HSA did spontaneously adhere to our treated surface in the bloodstream, the resulting HSA protein layer acted as a blocking layer to maintain the anti-thrombogenic property, in accordance with previous reports. Moreover, the HSA layer did not inhibit the adhesion of endothelial cells, which also corroborated previous reports. Our treated surface was positively charged so there was strong interaction with HSA, ensuring the stability of the protein layer so that anti-thrombogenicity was prolonged for a week in the in vivo pig study. Care must be taken with safety assessment, however, because endothelialization also occurs on the treated metal surface immediately after implantation^22^. Therefore, the exposed metal surface can be covered with neoendothelial cells. We are under investigation of histological sectioning and take lots of time for analysis of the endothelialization in pig study. We will report the in vivo analysis in the future.

Another group has produced anti-thrombogenic coatings by modifying surfaces with the Arg-Glu-Asp-Val peptide^33^. The oligopeptide does not have anti-thrombogenic properties itself but rather has a high affinity for the receptors of human endothelial cells, which can capture circulating vascular progenitor cells in the bloodstream^34^. According to their report, the circulating vascular progenitor cells spontaneously adhered to the oligopeptide-treated surface, conferring anti-thrombogenic properties. The anti-thrombogenic mechanism is not fully understood because it is not yet known how coagulation-related proteins are involved on the treated surface. However, autoregulation by the treated surface may retain anti-thrombogenicity. Therefore, our APTES coating provides a stable HSA protein layer in blood that spontaneously blocks protein adsorption and platelet adhesion, and has a different regulation mechanism from that of an anti-fouling polymer coating. The APTES coating also confers cell-adhesion properties.

## Conclusion

The APTES coating has both anti-thrombotic and cell adhesive properties, as assessed by a blood loop study, SEM observation, and cell adhesion analysis. Furthermore, the coating is safe, as evinced by short-term in vivo pig studies. Therefore, our APTES coating is particularly well-suited to stent applications and offers anti-thrombotic properties without hindering endothelialization in the clinical setting.

## Data Availability

The datasets used and/or analysed during the current study available from the corresponding author on reasonable request.
